# Omega 3 fatty acid improves sexual and erectile function in BPF-treated rats by upregulating NO/cGMP signaling and steroidogenic enzymes activities

**DOI:** 10.1038/s41598-023-45344-4

**Published:** 2023-10-23

**Authors:** Adeyemi Fatai Odetayo, Luqman Aribidesi Olayaki

**Affiliations:** 1 Physiology Department, University of Ilorin, Ilorin, Kwara State Nigeria; 2 Physiology Department, Federal University of Health Sciences, Ila Orangun, Osun State Nigeria

**Keywords:** Infertility, Sexual dysfunction

## Abstract

Bisphenol F (BPF) is an environmental pollutant that has been implicated in sexual dysfunction. Omega 3 fatty acid (O3FA), on the other hand, is an antioxidant with the ability to improve fertility indices. However, no study has explored the possible ameliorative effect of O3FA on BPF-induced sexual dysfunction. Thus, the effect of BPF and/or O3FA on male sexual performance was investigated. Male Wistar rats were randomized into 6 groups, corn oil-treated, O3FA low and high dose (100 and 300 mg/kg), BPF-treated, BPF + O3FA low and BPF + O3FA high dose. BPF significantly impaired male sexual competence, evidenced by a reduction in motivation to mate, prolonged mount, intromission and ejaculation latency, and post-ejaculatory index. Furthermore, a reduction in mount, intromission, and ejaculation frequency were observed. Also, BPF caused a decrease in gonadotropin releasing hormone, follicle stimulating hormone, luteinizing hormone, testosterone, nitric oxide (NO) cyclic guanosine monophosphate (cGMP), 3beta-hydroxysteroid dehydrogenase (3β-HSD), 17beta-hydroxysteroid dehydrogenase (17β-HSD), dopamine, and acetylcholine esterase. Furthermore, it was accompanied by a significant increase in prolactin and estrogen and poor pregnancy outcomes. These observed BPF-led alterations were abolished by O3FA administration. This study showed that O3FA ameliorates BPF-induced sexual dysfunction by upregulating NO/cGMP signaling and steroidogenic enzymes activities.

## Introduction

Sexual dysfunction (SD) is an impairment in sexual behavior and sexual stimulation that occurs as a result of abnormal or absence of psychological or physiological response to sexual stimuli. The term sexual dysfunction can be used as a general term for various symptoms including erectile dysfunction, loss of libido or desire, and failure to achieve sexual intercourse^1^. According to an epidemiological study reported by Chen et al.^1^, about 52% of the studied population that are 40–70 years old men were suffering from different types of sexual dysfunction, which resulted from different biological and psychological factors. Out of the estimated 15% occurrence of sexual disharmony in couples globally, about 50% were due to male factors, and this could be a result of the sensitivity of the male reproductive system to environmental pollutants.

Bisphenol analogs (BPs) such as bisphenol A (BPA), bisphenol F (BPF), and bisphenol S (BPS) have been classified as environmental pollutants or endocrine disruptors. These compounds are utilized extensively in the chemical, food, and pharmaceutical industries. It has been established that BPA, the bisphenol compound used the most frequently, is involved in the emergence of numerous human diseases, including male sexual dysfunction. Because of this, the use of BPA has been severely limited since 2010^2^. Concurrently, the industry has begun making widespread use of BPF, which serves as a significant alternative to BPA^2^. The presence of BPF in the environment is beginning to emerge as its use continues to increase. BPF is found in several environmental media, including indoor dust and water, in addition to being found in food, feeding bottles, cosmetics, and medical devices. In a review of numerous food items in the United States, BPF was shown to be the second most prevalent analog present in beverages, dairy products, fats and oils, fish and shellfish, meat, cereals, fruits, and vegetables^3^. Oral route is the major route of exposure to BPF but other routes include dermal and inhalation. Despite the continuous usage of BPF, there is little information on its effect on male sexual performance. The role of NO/cGMP signaling and steroidogenic enzymes in BPF-induced male sexual dysfunction is yet to be fully explored. Also, whether omega 3 fatty acid (O3FA) will ameliorate BPF-induced male sexual dysfunction is not known.

The penis is one of the external organs of the male reproductive system. The penis performs two major sexual functions which are erection and ejaculation. Penile erection involves a cascade of reactions that commences with the release of NO by the nerves and endothelial cells in the penis, and has been implicated in maintaining normal blood flow and male sexual performance^4,5^. A decline in the circulating NO has been linked with numerous disease conditions such as sexual dysfunction^6^. NO is synthesized from non-adrenergic/non-cholinergic parasympathetic nerves. It is released from both the cavernosal nerve ending and the endothelial cells of the penile artery upon stimulation from the spinal cord^7^. Once NO has been released, it stimulates a cascade of action, which leads to the relaxation of the smooth muscle of corpora cavernosa to stimulate penile erection. NO stimulates penile erection by positively modulating guanylate cyclase, which leads to the synthesis of cyclic guanosine monophosphate (cGMP).

Testosterone (T) is a major hormone responsible for male sexual functions^8^, and it is produced by the testis via the activities of steroidogenic enzymes. Steroidogenic Acute Regulatory Protein (StAR) translocates cholesterol into the inner mitochondria membrane of the testicular tissue, where it is being converted to pregnenolone before being acted on by 3beta-hydroxysteroid dehydrogenase (3β-HSD) to be converted to progesterone. The progesterone is then converted to a series of metabolic substrates before finally being converted into testosterone by 17beta-hydroxysteroid dehydrogenase (17β-HSD). Studies have shown that the upregulation of these enzymes increased the biosynthesis of testosterone. However, inhibition of these enzymes consequently leads to the suppression of the circulating level of testosterone^9^.

On the other hand, O3FA is a polyunsaturated fatty acid (PUFA) that can only be ingested exogenously through dietary supplement^10^. The three major O3FAs are docosahexaenoic acid (DHA), eicosapentaenoic acid (EPA) and alpha-linolenic acid (ALA). These three are required for a variety of functions, including growth, reproduction, vision, and the development of the brain^11^. While the data reporting the beneficial effect of O3FA on male sexual performance is lacking, it has been shown to have vasodilatory effect in different vascular beds^12^, and by extension, could cause vasodilation of smooth muscle of corpora cavernosa which can improve male sexual function via NO/cGMP signaling.

Findings from our previous studies revealed that cessation from BPF exposure did not completely reverse the observed BPF-induced sexual and testicular dysfunction^13,14^. Hence, this study was designed in an attempt to completely ameliorate the observed BPF-induced sexual dysfunction. Despite the beneficial effect of O3FA on male reproduction, no study has investigated its possible role on male sexual performance and pregnancy outcome. Hence, this study was designed to investigate the ameliorative effect of O3FA and its possible mechanisms of action on BPF-induced male sexual dysfunction.

## Methods and materials

### Experimental animals

One hundred and twenty Wistar rats (sixty males and sixty females) of similar weights were purchased from the Biochemistry Department, University of Ilorin. The animals were handled carefully based on the Guidelines for Laboratory Animal Care published by the National Institute of Health (NIH) and Animal Research: Reporting of In Vivo Experiments (ARRIVE) guidelines for reporting experimental findings were followed. The experimental protocol was under the guidelines of the National Research Council's for the Care and Use of Laboratory Animals, and ethical approval was obtained from the University of Ilorin Ethical Review Committee (UERC) (UERC/ASN/2022/2396). The animals were randomly divided into cages free of dirt under natural conditions and were given free access to feed and water ad libitum.

### Experimental design

The animals were allowed to acclimatize for 2 weeks and trained sexually as previously documented^15^, after which they were divided randomly into six groups (n = 10 rats per group); 0.5 ml of corn oil was given to the control group, while the animals in the positive control groups received 100 mg/kg (low dose) of O3FA (O3FA-L) and 300 mg/kg (high dose) of O3FA (O3FA-H), BPF treated rats were given 30 mg/kg of BPF. The rats that were treated with BPF + O3FA treated rats got 30 mg/kg BPF + low dose of O3FA (BPF + O3FA-L) and 30 mg/kg of BPF + a high dose of O3FA (BPF + O3FA-H). The dosage of BPF used in this study is similar to previously reported doses by^13,16,17^, while the dose of O3FA was earlier reported and used by^18^.

### Chemical preparation and administration

O3FA was procured from Gujarat Liqui Pharmacaps Pvt. Ltd, India, and each O3FA capsule contains eicosapentaenoic acid (EPA) and docosahexaenoic acid (DHA) in the ratio of 3:2. BPF was procured from Sigma-Aldrich, St. Louis, MO, USA (CAS: 620-92-8).

The dose of BPF was calculated based on the weight of each animal, dissolved in corn oil, and 0.5 ml of the solution, containing the appropriate calculated dose, was administered for each rat. BPF and O3FA were administered once daily via gavage for twenty-eight days. The oral route of administration was chosen to imitate the prominent human way of exposure. Over-night fasted animals were sacrificed 24 h after the last doses.

To evaluate sexual performance, the animals were made receptive by inducing estrous via the subcutaneous administration of 10 g/100 g BW of estradiol benzoate and 0.5 mg/100 g BW of progesterone forty-eight and four hours, respectively, prior to copulation^13,15,19^. Estrous status was determined based on the vaginal smear and the vaginal appearance at the time of examination. In other to confirm receptivity, female rats were introduced to male rats that were not a part of this research and were disengaged before mating^15^.

### Assessment of sexual performance

One male animal was placed per cage before introducing sexually receptive female rats (i.e., one male and one female rat per cage). Under dim light for 30 min, sexual performance was assessed and using a camcorder, sexual activities were recorded. The activities recorded such as mount frequency (MF), mount latency (ML), motivation to mate, intromission latency (IL), intromission frequency (IF), ejaculation frequency (EF), post-ejaculatory interval (PEI) and ejaculation latency (EL), were later viewed and scored based on the method of^15,19^. Briefly, Motivation to mate was scored as previously reported^19^:

- 0: no sexual activity
- 1: no interaction, rears and climbs on the chamber
- 2: sniffs the female rat
- 3: self- exploratory behavior such as grooming and sniffing of genitals
- 4: grooms female counterpart anywhere
- 5: rears and climbs sexually
- 6: pursues and sniffs the female rat
- 7: tries to mount but easily discouraged
- 8: mounts with an integrated deliberate manner and not easily discouraged
- 9: reflex and almost involuntary mount

ML: the time lag from when the female rat was introduced until the first mount when the rats achieved a copulatory position. MF: the number of times the male rats attained copulatory position but could not achieve intromission (vagina penetration). Mount is achieved when the male rat lifts its forebody to climb the female rat from the back and grasps her flanks with the forepaw. IL is the time between the introduction of the female till the first vagina penetration (intromission), while IF is the number of times the male rat attained intromission (vagina penetrations) but failed to ejaculate. Intromission is characterized by thrusting of the pelvic, followed by a rapid dismount. EL: is the time between the first intromission and ejaculation characterized by a deeper pelvic thrusting and gradual dismount, followed by a period of reduced sexual activity. EF number of ejaculations from the time of the introduction of the female animal to the male within 30 min. Post ejaculatory interval (PEI) was recorded as the time from the first ejaculation to the next copulatory behavior (mount or intromission).

### Measurement of progeny parameters and fertility indices

Fertility index, Success of Fertility, Litter weight, rate of survival at weaning and size of litter were obtained as described previously^15^.

Fertility success was determined as the number of female rats that are pregnant divided by the number of paired rats multiplied by 100.

Fertility index: Number of rats that are pregnant divided by number of mated rats multiplied by 100.

Size of litter: average amount of delivered progeny.

Weight of litter: average size of delivered progeny.

### Collection of samples

The overnight-fasted rats were sacrificed 24 h after the last administration under the influence of 4 mg/kg of xylazine and 40 mg/kg of ketamine via the intraperitoneal route^15^. Through cardiac puncture, blood samples were collected into heparinized containers. At 3000 rpm, the samples of blood were centrifuged for 5 min to obtain plasma which was used for biochemical analysis. Testes and penis were harvested and homogenized in cold phosphate buffer solution for biochemical assays. Penile cyclic guanosine monophosphate (cGMP) was estimated from the penile tissue while the testicular 3β-HSD and 17β-HSD were determined in the testicular tissue.

### Biochemical assays

Estimation of hormones was carried out according to the instructions provided by the ELISA kit manufacturers. Plasma gonadotropin releasing hormone (GnRH) was estimated as instructed by the manuturer (Melsin, China). Luteinizing hormone (LH), follicle-stimulating hormone (FSH), testosterone, estradiol, and prolactin (PRL) were estimated according to the manufacturer’s instruction (Bio-Inteco, UK). Acetylcholinesterase (AchE), cGMP (Elabscience, UK) and Dopamine (Adnova, US) were also assayed based on the instruction of the manufacturer using ELISA kits. The method of Ridnour et al.^20^ was used to estimate plasma nitric oxide (NO). Testicular 13β-HSD and 17β-HSD were estimated according to the method of Talalay^21^ and Jarabak et al.^22^. Briefly, for 3β-HSD, testicular tissue was homogenized, and the supernatant was carefully separated. 1 ml of the supernatant was mixed with 1 ml of 100 μmol sodium pyrophosphate buffer (pH 8.9), 30 μg of dehydroepiandrosterone in 40 μl of ethanol, and 960 μl of 25% BSA. The mixture was then incubated and 0.5 μmol of NAD was added. The absorbance was read spectrophotometrically at a wavelength of 340 nm using a blank as reference. For testicular 17β-HSD, 1 ml of the supernatant obtained from the testicular sample was mixed with 1 ml of 440 μmol sodium pyrophosphate buffer (pH 10.2), 40 μl of ethanol containing 0.3 μmol of testosterone, and 960 μl of 25% BSA. The mixture was incubated and 1.1 μmol of NAD was added in a U 2,000 spectrophotometer cuvette at 340 nm against a blank. For circulatory NO, a mixture of 100 μl of Griess reagent, 300 μl of a nitrate-containing testicular homogenate, and 2.6 ml of deionized water were incubated for 30 min at room temperature in a spectrophotometer cuvette. A blank was prepared by mixing 100 μl of Griess reagent and 2.9 ml of deionized water. The absorbance of the nitrate-containing sample was measured at 548 nm in relation to the reference sample.

### Statistical analysis

GraphPad PRISM 5 software (GraphPad Software, La Jolla, California, USA) was used in carrying out the statistical analysis with a one-way analysis of variance (ANOVA) and Tukey's post hoc test. Data were reported as mean ± standard deviation. Values of *P* below 0.05 were considered statistically significant.

## Results

### Sexual performance parameters

As shown in Table 1, BPF administration led to a significant reduction in motivation to mate (15.7%) when compared with the control groups, and the observed decrease was blunted by both the low and high doses of O3FA. In addition, treatment with low and high doses of O3FA prevented BPF-induced increased in ML, IL, and EL.

**Table 1:** Effect of O3FA on sexual performance parameters in BPF exposed rats.

Parameters	Control	O3FA-L	O3FA-H	BPF	BPF + O3FA-L	BPF + O3FA-H
Motivation to mate (score)	9.21 ± 0.26	9.12 ± 0.17	9.24 ± 0.20	7.75 ± 0.20^a,b,c^	9.09 ± 0.17^d^	9.12 ± 0.12^d^
ML (secs)	46.60 ± 3.85	47.00 ± 3.87	43.20 ± 3.11	108.80 ± 5.54^a,b,c^	72.20 ± 4.82^a,b,c,d^	53.00 ± 3.32^c,d,e^
IL (secs)	103.50 ± 2.74	98.33 ± 2.42^a^	95.17 ± 1.17^a^	136.00 ± 2.37^a,b,c^	123.20 ± 1.94^b,c,d^	103.80 ± 2.48^b,c,d,e^
EL (secs)	75.67 ± 1.75	71.00 ± 4.20	70.50 ± 5.75	120.50 ± 3.27^a,b,c^	86.67 ± 3.39^a,b,c,d^	76.50 ± 3.27^d,e^
MF (number)	16.80 ± 0.84	17.60 ± 1.83	17.00 ± 2.00	9.80 ± 2.17^a,b,c^	15.00 ± 1.23^d^	16.60 ± 1.67^d^
IF (number)	17.00 ± 1.58	18.00 ± 0.71	16.40 ± 1.14	11.40 ± 1.14^a,b,c^	15.80 ± 1.30^d^	16.60 ± 0.89^d^
EF(number)	11.00 ± 1.41	11.17 ± 1.17	14.00 ± 1.67	4.17 ± 0.75^a,b,c^	8.50 ± 0.84^a,b,c,d^	10.50 ± 1.05^c,d^
PEI (sec)	151.60 ± 6.35	140.40 ± 5.98^a^	129.60 ± 5.98^a,b^	210.60 ± 3.05^a,b,c^	148.60 ± 6.43^,c,d^	144.40 ± 3.58^d^

*ML* mount latency, *IL* intromission latency, *EL* ejaculatory latency, *MF* mount frequency, *IF* intromission frequency, *EF* ejaculatory frequency, *PEI* post-ejaculatory interval.

^a^*p* < 0.05 versus control, ^b^*p* < 0.05 versus omega 3 fatty acid low dose (O3FA-L), ^c^*p* < 0.05 omega 3 fatty acid high dose (O3FA-H), ^d^*p* < 0.05 versus bisphenol f (BPF), ^e^*p* < 0.05 versus BPF + O3FA-L at *p* < 0.05 using one-way analysis of variance (ANOVA) followed by Tukey’s post hoc test for pairwise comparison.

Furthermore, BPF exposure led to a significant reduction in MF (41.7%), IF (32.9%), and EF (62%) compared with the vehicle treated and O3FA treated control groups. Co-administration of BPF with O3FA significantly prevented BPF-induced reduction in MF, IF, and EF.

### Hormonal changes

Although BPF did not lead to a significant decrease in plasma GnRH compared with the control groups. However, BPF exposure brought about a significant reduction in plasma LH (*p* < 0.0001), FSH (*p* < 0.001), and testosterone (*p* < 0.0001). While the observed decreased following BPF exposure was abolished by co-administration of BPF with both the low and high dose of O3FA, treatment with O3FA-H is more effective in ameliorating the BPF-induced hormonal imbalance.

Also, BPF exposure significantly increased the level of plasma estrogen (*p* < 0.0001) and prolactin (*p* < 0.0001) compared with the corn oil and O3FA treated control groups. Although the observed increase in plasma prolactin was negated by the co-administration of BPF with both the low and high dose of O3FA, the observed ameliorating effect was more pronounced with the high dose of O3FA treatment (Table 2).

**Table 2:** Effect of O3FA on Reproductive hormones in BPF exposed rats.

Parameters	Control	O3FA-L	O3FA-H	BPF	BPF + O3FA-L	BPF + O3FA-H
GnRH (mIU/mL)	5.746 ± 0.370	5.771 ± 0.824	5.812 ± 0.887	5.680 ± 0.577	5.756 ± 0.714	6.132 ± 0.446
LH (mIU/mL)	6.125 ± 0.289	6.178 ± 0.659	6.131 ± 1.130	2.500 ± 0.718^a,b,c^	4.313 ± 1.003^a,b,c,d^	5.806 ± 0.836^d^
FSH (mIU/mL)	4.123 ± 0.145	4.080 ± 0.541	3.845 ± 0.602	2.677 ± 0.332^a,b,c^	3.457 ± 0.528	4.046 ± 0.399^d^
Testosterone (ng/mL)	2.336 ± 0.185	2.809 ± 0.170^a^	2.824 ± 0.129^a^	1.229 ± 0.151^a,b,c^	2.068 ± 0.154^b,c,d^	2.550 ± 0.111^d,e^
Estradiol (ng/mL)	5.141 ± 0.130	5.100 ± 0.239	4.912 ± 0.196	6.998 ± 0.347^a,b,c^	5.089 ± 0.310^d^	4.956 ± 0.334^d^
Prolactin (ng/mL)	0.619 ± 0.053	0.646 ± 0.077	0.644 ± 0.081	1.475 ± 0.081^a,b,c^	0.594 ± 0.078^d^	0.602 ± 0.060^d^

*GnRH* gonadotropin releasing hormone, *LH* luteinizing hormone, *FSH* follicle stimulating hormone.

^a^*p* < 0.05 versus control, ^b^*p* < 0.05 versus omega 3 fatty acid low dose (O3FA-L), ^c^*p* < 0.05 omega 3 fatty acid high dose (O3FA-H), ^d^*p* < 0.05 versus bisphenol f (BPF), ^e^*p* < 0.05 versus BPF + O3FA-L at *p* < 0.05 using one-way analysis of variance (ANOVA) followed by Tukey’s post hoc test for pairwise comparison.

### Dopamine and AChE

**Figure 1:**
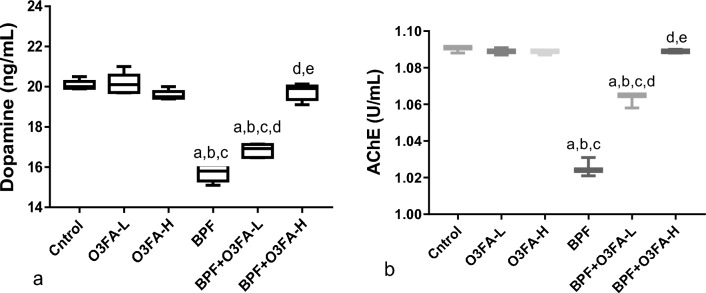
Effect of and omega 3 fatty acid (O3FA) on (**a**) dopamine (**b**) acetyl cholinesterase (AChE) in bisphenol F (BPF) exposed rats. ^a^< 0.05 versus control, ^b^*p* < 0.05 versus omega 3 fatty acid low dose (O3FA-L), ^c^*p* < 0.05 omega 3 fatty acid high dose (O3FA-H), ^d^*p* < 0.05 versus bisphenol f (BPF), ^e^*p* < 0.05 versus BPF + O3FA-L (n = 5) at *p* < 0.05 using one-way analysis of variance (ANOVA) followed by Tukey’s post hoc test for pairwise comparison.

BPF significantly decreased circulating dopamine (*p* < 0.0001) and AChE (*p* < 0.0001) compared with the vehicle and O3FA control rats (Fig. 1). Co-administration of BPF with O3FA led to a significant increase in dopamine and AChE compared with BPF exposed rats. Also, co-administration of high dose O3FA with BPF proved to be more effective than O3FA-L with BPF.

### NO/cGMP signaling

**Figure 2:**
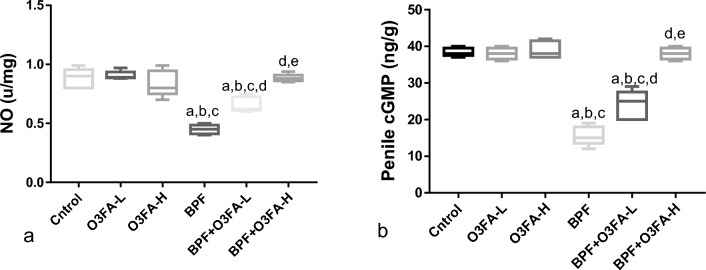
Effect of omega 3 Fatty Acid (O3FA) on (**a**) nitric oxide (NO) (**b**) cyclic guanosine monophosphate (cGMP) in bisphenol F (BPF) exposed rats. ^a^*p* < 0.05 versus control, ^b^*p* < 0.05 versus omega 3 fatty acid low dose (O3FA-L), ^c^*p* < 0.05 omega 3 fatty acid high dose (O3FA-H), ^d^*p* < 0.05 versus bisphenol f (BPF), ^e^*p* < 0.05 versus BPF + O3FA-L (n = 5) at *p* < 0.05 using one-way analysis of variance (ANOVA) followed by Tukey’s post hoc test for pairwise comparison..

Exposure to BPF significantly reduced both circulating NO and penile cGMP when compared with the control groups. While the co-administration of both the low and high dose of O3FA with BPF abolished the observed decrease, it was more pronounced in the rats with high dose O3FA and BPF co-administration (Fig. 2).

### Steroidogenic/androgenic enzymes

**Figure 3:**
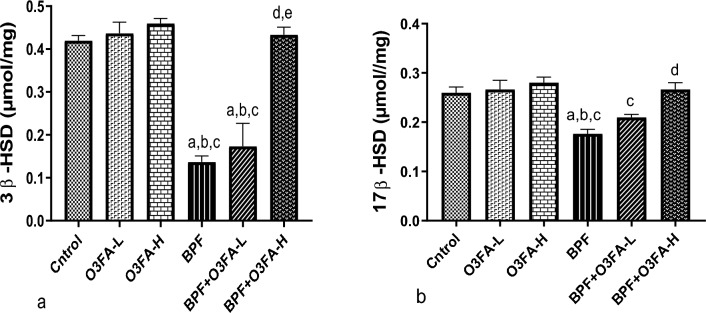
Effect of omega 3 fatty acid on testicular (**a**) 3 beta-hydroxysteroid dehydrogenase (3β-HSD) (3 β-HSD) (**b**) 17 beta-hydroxysteroid dehydrogenase (17 β-HSD) in BPF exposed rats. ^a^*p* < 0.05 versus age-matched control, ^a^*p* < 0.05 versus control, ^b^*p* < 0.05 versus omega 3 fatty acid low dose (O3FA-L), ^c^*p* < 0.05 omega 3 fatty acid high dose (O3FA-H), ^d^*p* < 0.05 versus bisphenol f (BPF), ^e^*p* < 0.05 versus BPF + O3FA-L at (n = 5) *p* < 0.05 using one-way analysis of variance (ANOVA) followed by Tukey's post hoc test for pairwise comparison.

The observed decrease in testicular 3β-HSD and 17β-HSD following BPF exposure was abolished by low and high doses of O3FA treatment (Fig. 3). However, animals treated with a high dose have improved steroidogenic enzymatic activities compared to those treated with a low dose.

### Fertility index and success

As indicated in Table 3, all the ten animals in the control groups and BPF + O3FA-H were able to mate. Nine animals conceived, indicating that the fertility success was 90%, and the fertility index was 90%. In the BPF treated and BPF + O3FA-L, nine animals mated and eight were able to conceive, indicating that reproductive success and the index were 80 and 87.5%, respectively.

**Table 3:** Effect of O3FA on fertility indices and progeny parameters in BPF exposed rats.

Parameters	Control	O3FA-L	O3FA-H	BPF	BPF + O3FA-L	BPF + O3FA-H
Fertility success (%)	90.00	90.00	90.00	80.00	80.00	90.00
Fertility index (%)	90.00	90.00	90.00	87.50	87.50	90.00
Litter size (number)	9.02 ± 0.22	9.07 ± 0.27	9.17 ± 0.24	8.93 ± 0.33	9.05 ± 0.25	9.12 ± 0.12
Litter weight (grams)	6.27 ± 0.22	6.24 ± 0.42	6.11 ± 0.20	6.02 ± 0.13	6.05 ± 0.15	6.07 ± 0.16
Survival at weaning (%)	98.00	98.00	98.00	90.00	96.00	98.00

### Pregnancy outcome

A decrease in litter size and weight was observed in offspring sired by BPF-exposed rats compared to their counterparts fathered by control and O3FA-treated animals (Table 3).

Out of the 48 pups sired by vehicle-treated rats, 47 (98%) survived weaning, while 49 (98%) out of the 50 pups fathered by low and high doses O3FA treated rats survived after three weeks of birth. Furthermore, 90% of the pups sired by animals exposed to BPF alone survived weaning (i.e., 39 out of 43). In addition, BPF + O3FA-L and BPF + O3FA-H administered rats fathered 45 and 47 pups, respectively, and while 43 (96%) pups survived in the BPF + O3FA-L, 46 (98%) pups survived weaning in the BPF + O3FA-H group.

## Discussion

Male sexual function is a composite of various physiological processes and an important contributing factor to a good quality of life. Maintaining optimal male sexual function depends on coordinating the various body systems, such as the nervous, endocrine, cardiovascular, and reproductive systems^1,23^. Once any of the systems mentioned above or the physiological components are disrupted, normal male sexual function quality will also be affected. The male sexual function consists of the whole process of males’ sexual activities, such as sexual desire/libido, erection of the penile tissue, and sexual activities (vagina penetration and ejaculation)^24^, and any impediment in any of these links is referred to as sexual dysfunction.

Sexual dysfunction is a complex physiological process that can be distorted by various pathological conditions such as endothelial dysfunction, hormonal imbalance, and neurological disorders^1^. Numerous allopathic drugs have been designed for treating sexual dysfunction, but these drugs are not without their side effects. Based on this, researchers are intensifying their efforts to search for a natural supplement with fewer side effects that are readily available and affordable. Hence, this study was designed to probe the impact of BPF on sexual dysfunction and pregnancy outcomes by exploring the mechanism underlying male sexual function. Also, the possible ameliorative effect of O3FA on male sexual dysfunction was investigated. The findings from this research will establish the various mechanisms of action for BPF-induced sexual dysfunction and provide another therapeutic intervention.

In animal models, male sexual desire and competence are determined by estimating the lag time (latency) and number (frequency) of mounts, intromission and ejaculations when paired with a female receptive partner^15^. In this study, the observed decrease in motivation to mate, MF, IF and EF, and the extended ML, IL, EL, and PEI following BPF exposure indicates the sexual inhibitory effects of the chemical on male sexual function. Furthermore, the observed difference in these parameters after O3FA treatment suggests the therapeutic effect of omega 3 fatty acid in male sexual dysfunction.

According to Yakubu and Akanji^19^, mount and intromission frequencies are indices of libido and sexual potency. Furthermore, IF is an indication of erection efficiency and effectiveness of ejaculatory reflexes^25^. Also, the ML and IL are inversely related to sexual arousal^26^, i.e., the higher the latencies, the lower the sexual arousal and vice versa. In addition, the PEI is an index of sexual vigour and determines the rate of reinstation from tiredness after an episode of sexual activity^27^.

In this study, all the aspects of the studied sexual activities adversely affected by BPF exposure were ameliorated by O3FA treatment. O3FA improved libido, sexual potency, and erection efficiency (evidenced by an increase in MF and IF and a decrease in ML and IL) following BPF exposure. Furthermore, O3FA improved ejaculatory reflex stimulation, an important indicator of sexual dysfunction, by decreasing BPF-induced prolonged EL^28^.

Although sexual performance parameters indicate erection efficiency, penile erection is influenced by NO/cGMP signaling^29^, an endothelial function. NO is one of the major active secretions of the endothelial cells that line the blood vessels. It is a nonadrenergic and noncholinergic neurotransmitter with vasodilatory function. NO is produced from the penile smooth muscles, and it is activated once there is a sexual stimulus by the dopamine-oxytocin-NO pathway^30^. NO enters the smooth muscle cells of the corpus cavernosum, where it acts on the guanosine cyclase to produce cGMP from guanosine triphosphate (GTP). The production of cGMP leads to vasodilation, which promotes penile erection. The observed downregulation of NO/cGMP signaling suggests that BPF induces erectile dysfunction by disrupting the signaling pathway. Apart from the role of this pathway on penile erection, NO has also been implicated in regulating hormonal secretions from the testis^1^, which are responsible for maintaining sexual functions; it is, however, tempting to conclude that the observed reduction in libido, sexual arousal, and erection efficiency following BPF exposure could be a result of hormonal imbalance via a NO-dependent mechanism. In contrast, O3FA administration restored the observed downregulation of NO/cGMP signaling following BPF exposure. It is important to note that the discovery from this study that O3FA restored the disruption of NO/cGMP signaling is novel, suggesting its therapeutic effectiveness in treating erectile dysfunction.

The male sexual function requires both the peripheral and central nervous systems to be intact^31^. Researchers have established the roles of monoamine neurotransmitters on erectile functions using animal models, while human studies have also suggested their roles in sexual desire^32^. Dopamine is a monoamine neurotransmitter, and it has been established to be required for motor activities that are important for sexual performance^33^. Also, dopamine has been shown to trigger penile erection via its action on the oxytocinergic neurons that are present in the paraventricular nucleus (PCN) of the hypothalamus and also in the pro-erectile sacral parasympathetic nucleus of the spinal cord (SPC)^34^. Therefore, it is plausible to infer that the BPF-induced sexual dysfunction could be due to, at least in part, BPF-induced decline in dopamine.

Additionally, the observed decrease in AChE could account for the BPF-induced sexual dysfunction. The finding from this study that BPF decreased AChE is similar to previous studies that reported similar observations following BPF exposure^35^ and its analog^36,37^. Although, it is expected that a decline in AChE should improve sexual function since AChE activities have been shown to breaks down or hydrolyzes acetylcholine (ACh), which is also responsible for penile erection^38^. In addition, the presence of ACh also stimulates dopamine release^39–41^. This suggests that the presence of AChE could inhibit the release of dopamine via its inhibitory effect on ACh. However, the decline in AChE in this study is accompanied by a decline in dopamine, suggesting an independent pathway. The observed dopamine could result from the observed BPF-induced hyperprolactinemia since the inverse relationship between dopamine and prolactin has been previously established^42^. On the other hand, BPF-induced decline in AChE activities could be a result of oxidative stress^37,43^ since hydroxyl radicals are involved in AChE inhibition^44^. The observed BPF-induced reduction in AChE will eventually leads to the accumulation of ACh which has also been shown to increase oxidative damage^45,46^. Furthermore, increase in ACh also inhibit GnRH secretion which in turn leads to a decrease in libido by impairing the activities of the HPG axis^47^. The result from this study that O3FA blunted BPF-induced reduction in dopamine agrees with the study of Chalon et al.^48^ that supplementation of O3FA increased the level of dopamine and D2 receptor binding. It is also possible that the ameliorative effect of O3FA on BPF-induced reduction in AChE activities is via its antioxidative properties^49^, since BPF-induced AChE inhibition has been linked with oxidative stress.

The endocrine system tightly regulates normal sexual function by forming a closed-loop feedback network. The hypothalamus releases GnRH to stimulate the secretion of FSH and LH from the pituitary gland, which then acts on the gonads to produce testosterone and estrogen. Testosterone production can be impaired once there is a disruption in the hypothalamic-pituitary–gonadal axis. Testosterone is essential for libido, and its deficiency has been implicated in sexual dysfunction^1^. The observed disruption in the hypothalamic-pituitary–gonadal axis following BPF administration could explain the observed sexual dysfunction. Furthermore, the findings from this study that O3FA ameliorates BPF-induced endocrine dysfunction agree with the study of Akhigbe et al.^10^ that reported a similar effect of O3FA on LH, FSH, and testosterone.

In addition, the observed hyperprolactinemia following BPF exposure contributes to the observed sexual dysfunction in this study. Prolactin has been shown to increase following orgasm^50^, inhibiting the surge in sexual motivation and arousal to create a post-orgasmic refractory period. Hyperprolactinemia has been implicated in male sexual dysfunction^51^, and the possible mechanism of action is still under debate. The observed increase in prolactin and decrease in testosterone following BPF exposure suggests that prolactin impaired male sexual function via a testosterone dependent mechanism. Decreased circulating testosterone has been linked with hyperprolactinemia, which inhibits LH secretion^52^, leading to further decline in circulating testosterone. Also, hyperprolactinemia can impair testosterone 5α reduction into dihydrotestosterone^53^, which accounts for testosterone function. Furthermore, BPF has also been established to have weak estrogenic activities^54^. BPF exposure might act as an estrogen agonist by binding with estrogen receptors to increase estrogenic activities. Previous studies have linked estrogen with an increase in prolactin secretion by either upregulating prolactin receptor gene expression and prolactin-secreting cells or downregulating the expression of dopamine receptors^42,55^. Furthermore, the observed decrease in prolactin following O3FA treatment of BPF-induced hyperprolactinemia agrees with Emsley et al.^56^, that observed that eicosapentaenoic acid (EPA) decreased the level of circulating prolactin in psychiatric patients.

The testis containing Leydig cells is the major male reproductive organ or gonad located in the scrotum. The main function of Leydig cells is to produce testosterone via steroidogenesis. The key enzymes in testicular steroidogenesis are the 3β and 17β-HSD^57^, and disruption in their activities could lead to a decline in testosterone production. The observed ameliorative effect of O3FA on 3β and 17β-HSD in BPF-induced distortion in these androgenic enzymatic activities could be the reason for the observed O3FA-induced increase in testosterone following BPF exposure.

## Conclusion

In conclusion, this study suggests O3FA ameliorates BPF-induced sexual dysfunction by upregulating NO/cGMP signaling and steroidogenic enzymatic activities.

## Data Availability

The data that support the findings of this study are available on request from the corresponding author.
